# Self-potential dataset for mapping groundwater flow patterns in the Chaîne des Puys (Auvergne, France)

**DOI:** 10.1016/j.dib.2023.109533

**Published:** 2023-08-30

**Authors:** Cyril Aumar, Philippe Labazuy, Solène Buvat, Emmanuel Delage

**Affiliations:** a Université Clermont Auvergne, CNRS, IRD, OPGC, Laboratoire Magmas et Volcans, F-63000 Clermont-Ferrand, France; b Université Clermont Auvergne, Observatoire de Physique du Globe de Clermont-Ferrand, F-63000, Clermont-Ferrand, France; c UMR 6249 CNRS Chrono-environnement, Université de Franche-Comté, F-25030 Besançon, France; d CNRS, SIGMA Clermont, Institut de Chimie de Clermont-Ferrand, Université Clermont Auvergne, F-63000 Clermont-Ferrand, France

**Keywords:** Hydrogeophysics, Volcanic aquifers, CAPRICE project, Drinking water supply, Geophysical survey

## Abstract

The present Self-Potential (SP) dataset acquired in the Chaîne des Puys is the result of four decades of measurements carried out by master's students, PhD students, researchers, and engineering offices under the auspices of the *Laboratoire Magmas et Volcans (LMV) and the Observatoire de Physique du Globe de Clermont-Ferrand (OPGC)*. Acquired in the 1980s by Maurice Aubert and his collaborators (e.g. [1], [2], [3]), this Self-Potential dataset was completed as part of the CAPRICE project focused on the hydrosystem of the Chaîne des Puys. The methodology and equipment used for data acquisition has remained unchanged since the first measurement in 1987. As a result, this dataset compiles more than 20,000 SP measurements and covers an area of almost 200 km².

The SP data are intended to serve as the basis for geological models, coupled with geological and other geophysical data, according to the method described in *Aubert and Atangana, 1996*. After interpolation, SP data can be used to identify preferential groundwater flow paths and to delineate the surface of hydrogeological watersheds. As indicated in the literature, they also be used to identify possible recharge zones or areas of permeability contrast.

Specifications Table

**Table:** 

Subject	Geophysics
Specific subject area	Self-Potential data
Type of data	Csv files GIS files (.shp and .qml)
How data were acquired	Self-Potential data were acquired using a high-impedance voltmeter (100 MΩ) and a sensitivity of 0.1 mV and a pair of nonpolarizable electrodes (Cu/CuSO4 electrodes). The georeferencing of the data was done using a handheld GPS (Garmin 66st). The altitude was calculated from a Digital Terrain Model with a resolution of 0.5 m and an accuracy of 0.1 m
Data format	Raw data Filtered data Processed data Referenced data
Description of data collection	Acquiring a SP profile involves fixing a first electrode as a reference electrode and using a second electrode as a roving electrode to sample the electric potential at the ground surface every 30 meters with a high impedance voltmeter (designed by OPGC). In our case, acquiring a measurement point involves digging a hole 20 cm deep, taking a GPS point (X; Y) and measuring the electric field using the mobile electrode. Measurements are included on database after that each SP measure are fixed to a single reference electrode, called the regional reference with a SIG processing using QGIS.
Data source location	This dataset comprises 22,512 SP measurements, of which 10,655 measurements were taken between 1987 and 2000 and 11,857 measurements as part of the CAPRICE project. This mapping of the SP signal mapping covers 200 km² of the 240 km² out of the Chaîne des Puys field.
Data accessibility	Repository name: OPGC and Zenodo; Data Identification number: OPGC: 864924BE-6925-4643-9337-96FC04C1A3B2 Zenodo: 10.25519/ZGHC-N543 Direct link to data: OPGC; https://doi.org/10.25519/ZGHC-N543 Zenodo: https://zenodo.org/record/8160397

## 1 Value of the Data

• This dataset is original in terms of both the density of SP measurements and the surface area covered.
• This dataset is useful for determining the main groundwater circulation axes in the volcanic aquifers of the Chaîne des Puys).
• This dataset is freely accessible on the OPGC platform. It is open to all uses related to the geology or hydrogeology of the Chaîne des Puys
• As part of the CAPRICE project, this dataset was used to model subsurface geological structures (< 300m depth) associated with geological data (boreholes and cross-sections).
• Self-Potential measurements can be used in conjunction with other geophysical data (electrical resistivity, magnetism or seismic) to perform cross-correlations or joint inversions.

## 2 Objective

Self-Potential measurements have long been used in the hydrogeological context of the Chaîne des Puys. Since the 1990s and the work of Aubert and Atangana [[1], [3]], a GIS database has been created to compile all SP data available on a local scale [2]. As part of the CAPRICE project (2018-2023), this dataset has been completed, homogenized and formatted in order to share this valuable dataset.

The main objective of this geographically extensive dataset is to produce an SP map of the Chaîne des Puys to (1) locate the main groundwater flow paths and (2) use this SP map as a basis for modeling the pre-volcanic topography of the Chaîne des Puys as a geological model.

## 3 Data Description

The dataset is available in both spreadsheet (.csv) and GIS formats. There are nine data files in the repositories with six (.SHP, .SHX, .QMD, .PRJ, .CPG, .DBF) of which correspond to SIG file. The .QML file is a layer setting file use in QGIS to formatting the dataset layer. In addition, a .csv file is available with a .docx document that present the dataset construction and use.

The attributes table in SIG format has the same format as the .csv file (Table 1) and is divided into 9 columns: the first is an index number from 1 to 22513, the next three are geographical coordinates (XYZ) in RGF93-Lambert 93 format. This is followed by the date of acquisition (between 1987 and 2022), the bibliographic reference number of the data origin, the sectorization of the data (North, South and Central Chaîne des Puys) and finally the SP value.

**Table 1:** header of the dataset in .CSV format.

Num	X	Y	Z	Date	RAPPORT	Regional loc	PS
1	694228,05	6527934,24	899	01/06/1987	Rapport 6	Nord ChdP	-155
2	694203,08	6527934,45	899	01/06/1987	Rapport 6	Nord ChdP	-171
3	694180,01	6527924,65	898	01/06/1987	Rapport 6	Nord ChdP	-169

All the SP data presented are referenced to an absolute reference where the SP signal is equivalent to 0 mV. SP values are therefore potential differences between the measurement point and this absolute reference. For this SP dataset, the regional reference is measurement number 8656 located in the northern part of the Chaîne des Puys.

**Fig. 1:**
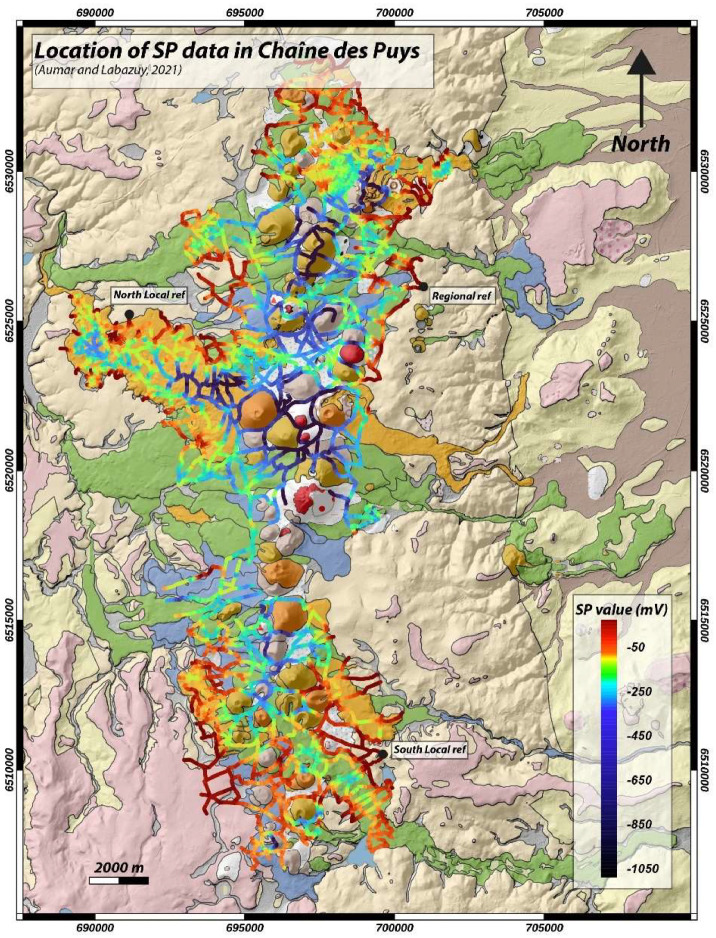
Geological map of the Chaîne des Puys with the SP dataset represented by colored dots as a function of the SP signal intensity between regional reference and the measuring point.

This dataset comprises 22,512 SP measurements, of which 10,655 measurements were taken between 1987 and 2000 and 11,857 as part of the CAPRICE project. This SP signal mapping covers 200 km² of the 240 km² Chaîne des Puys field (Fig. 1).

The bibliographic references from which the SP data in each report originate are included in the file on the OPGC repositories [4]. The altitude of each point (Z value) is extracted by GIS from a Digital Terrain Model with a resolution of 0.5 m (and an accuracy of +/- 0.1 m) from XY coordinates acquired with a conventional GPS (Garmin 66st, accuracy of about 2 m).

## 4 Experimental Design, Materials and Methods

a) SP principles in hydrogeology

The Self-Potential (SP) method is a passive, non-intrusive geoelectrical method for measuring the Earth's electrical field at the ground surface. Large- and small-scale Self-Potential anomalies are associated with physical processes on at the microscopic scale. In hydrogeological prospecting, the contribution of groundwater flow is mainly due to the stream potential effect [5], [6]. All minerals surface in contact with an aqueous solution become electrically charged [7]. The exchange of electric charge between minerals and fluid takes place at an interface known as the electric double layer (EDL, [8], [9], [10]). As the water contained in the porous medium flows, the displacement of this excess charge generates a current density known as the streaming current density. This results in electromagnetic disturbances in Maxwell's equations and hence in an electrical field component.

Other phenomena can create SP signals and coexist in the same zone, such as the thermoelectric effect [11], [12] or the electrochemical effect (redox potential, [13]).

b) *SP in the Chaîne des Puys*

**Fig. 2:**
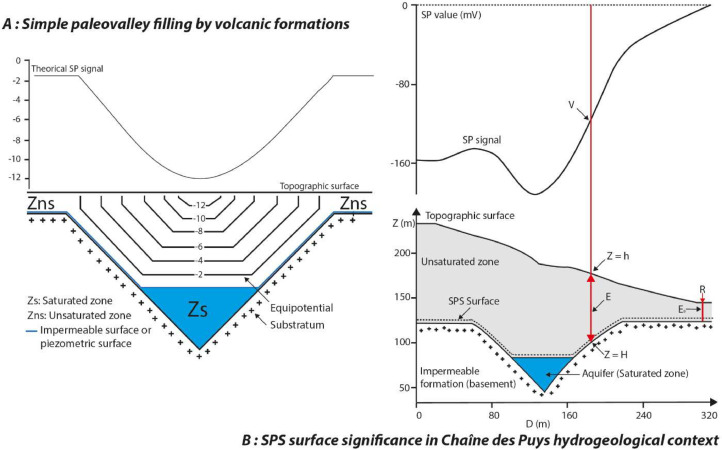
Some examples of SP profile in the context of the Chaîne des Puys. (A) Typical case of an SP signal in the context of a paleo-valley filled with volcanic formations and a flat topography. (B) Representation of a SP signal in a particular context with the different numerical parameters entering in the SPS modeling [[1], [3]].

Although several causes have been put forward to explain SP signal [1], [14], streaming potential is the main phenomenon to be considered in the Chaîne des Puys [14], coupled with a topographic effect [15], [16]. The SP value generally decreases with decreasing altitude due to the reduction in the distance traveled by water between the topographic surface and the saturated zone. Thus, the intensity of the SP signal is a function of the thickness of the unsaturated zone (see example of a flat topography, Fig. 2a). It can be observed on an active volcano such as Piton de la Fournaise, El Misti or others [17], [18] and also for a monogenic volcanic field such as the Chaîne des Puys (France, [1]) or Garrotxa (Spain, [19]).

In this context, the unsaturated zone is relatively thin, and the SP signal is therefore low (tending towards zero). Conversely, in areas where the unsaturated zone is thick, as in the case of a volcanic edifice, the SP signal is high. In the absence of a saturated zone, the SP signal corresponds to the distance between the topographic surface and the first impermeable surface, which in the case of Chaîne des Puys corresponds to the crystalline basement [1].

To image the basement of the unsaturated zone, [1] defined a geophysical surface, called the SPS surface, calculated from SP values and topographic data (Fig. 2b). The *H*(*x,y*) elevation of this surface is given by Eq. (1):

(1) $$H\left(x,y\right)=h\left(x,y\right)-\frac{V\left(x,y\right)}{K}-E_{0}$$

where *h*(*x,y*) is the elevation at the measurement point, *V*(*x,y*) is the negative Self-Potential anomaly, K is a coefficient in Volts per meter and *E*_0_ represents the thickness of the unsaturated zone at the reference R. K and *E*_0_ must be estimated or calculated from borehole data [1].

c) *Method and field acquisition strategy*

To acquire SP data at the ground surface, we used nonpolarizable electrodes, such as Cu/CuSO4 electrodes (Aubert type). The electrical potential difference is measured by connecting the electrodes to a voltmeter using an insulated wire: in our case, a 500 m long wire with a diameter of 2.4 mm. The voltmeter used in this study was manufactured at OPGC in the 1980s, with a high-impedance voltmeter (100 MΩ) and a sensitivity of 0.1 mV.

The map shown in Fig. 1 is based on a compilation of several SP profiles made at different times. Acquiring a SP profile involves fixing a first electrode as a reference electrode and using a second electrode as a roving electrode to sample the electric potential at the ground surface every 30 m. In our case, acquiring a measurement point involves digging a hole 20 cm deep, taking a GPS point (X; Y) and measuring the electric field using the mobile electrode. In the context of the Chaîne des Puys, the use of bentonite or salt water is not necessary, as the contacts are of good quality throughout the year. At the beginning, middle and end of each acquisition session, the electrode-ground contact resistance was tested to ensure that measurements were carried out in a homogeneous environment.

In agreement with the methodology described in Barde-Cabusson et al. [20] and Revil et al. [18], data acquisition is carried out by forming closed loops to create an interconnected mesh of profiles. Once the loops are closed, all data must be set to the same reference. It is sometimes necessary to make a correction when closing the loop, due to the difference in measurement between the first and last measurement at the same point (material drift, variation in contact resistance, changes in environmental parameters); this is known as the closure deviation.

**Fig. 3:**
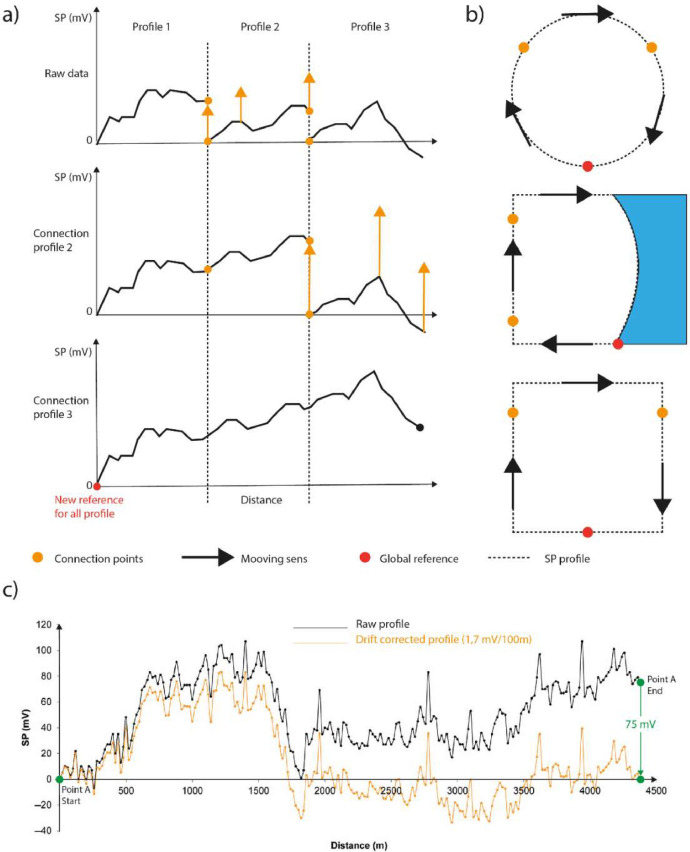
(a) connection of three PS profiles on the same reference;(b) loop and referencing scheme in different cases (loop, volcanic island or sum of profiles); (c) example of a drift correction on the same point (modified from Barde-Cabusson et al. [20]).

The final step is to fix all the interconnected data (set of loops) to a single reference electrode, called the regional reference. This methodology is well described in Barde-Cabusson et al. [20] and illustrated in Fig. 3, modified on the basis of this study.

## Ethics Statements

This work does not involve the use of human subject, does not involve animal experiment and does not involve data collected from social media platforms.

## CRediT authorship contribution statement

**Cyril Aumar:** Investigation, Formal analysis, Visualization, Writing – original draft, Writing – review & editing. **Philippe Labazuy:** Investigation, Supervision, Writing – review & editing. **Solène Buvat:** Investigation, Writing – review & editing. **Emmanuel Delage:** Data curation.

## Data Availability

Self-Potential dataset for mapping groundwater flow patterns in the Chaîne des Puys (Original data) (Zenodo).
